# Emerging Trends in International Law Concerning Global Infectious Disease Control^1^

**DOI:** 10.3201/eid0903.020336

**Published:** 2003-03

**Authors:** David P. Fidler

**Affiliations:** * Indiana University School of Law, Bloomington, Indiana, USA

**Keywords:** Infectious diseases, international law, International Health Regulations, World Trade Organization, global governance, globalization, human rights, access to drugs, perspective

## Abstract

International cooperation has become critical in controlling infectious diseases. In this article, I examine emerging trends in international law concerning global infectious disease control. The role of international law in horizontal and vertical governance responses to infectious disease control is conceptualized; the historical development of international law regarding infectious diseases is described; and important shifts in how states, international institutions, and nonstate organizations use international law in the context of infectious disease control today are analyzed. The growing importance of international trade law and the development of global governance mechanisms, most prominently in connection with increasing access to drugs and other medicines in unindustrialized countries, are emphasized. Traditional international legal approaches to infectious disease control—embodied in the International Health Regulations—may be moribund.

Globalization creates challenges for infectious disease policy (*1*–*3*). These challenges are horizontal and vertical in nature. Horizontal challenges constitute problems that arise between states from global microbial traffic (*4*). Vertical challenges, such as inadequate surveillance capacity (*5*), are problems countries face inside their territories that require responses within states*.* States cannot handle horizontal or vertical challenges without cooperating with each other. Unilateral efforts have limited impact when the source of the problem is beyond national jurisdiction (*6*). Similarly, unindustrialized countries need assistance to improve domestic public health (*7*). International cooperation mechanisms, including international law, are crucial to respond to both types of challenges. I examine the role international law plays in responses to horizontal and vertical challenges, analyze the historical development of international law in this area, and explore emerging trends in international law on infectious diseases that depart from traditional patterns.

## Governance Responses to Globalization

The challenges globalization presents for infectious disease policy require governance responses. For horizontal challenges, the response of the government focuses on interstate cooperation to minimize disease exportation and importation. Vertical challenges require strategies that reduce disease prevalence through improved domestic public health.

The state constitutes the key actor in infectious disease governance. Public health is a “public good,” which the public sector must produce because private actors lack sufficient incentives or resources (*7*). Governance responses to globalization occur at national, international, and global levels (Figure). National governance occurs when a state acts within its own territory to respond to globalization. International governance involves states cooperating to confront globalization challenges and often creates norms, rules, and institutions (i.e., regimes) to facilitate cooperation. Global governance involves not only states and international organizations but also nonstate actors, such as multinational corporations and nongovernment organizations (*8*,*9*), whose participation becomes critical to the success of governance efforts.

**Figure:**
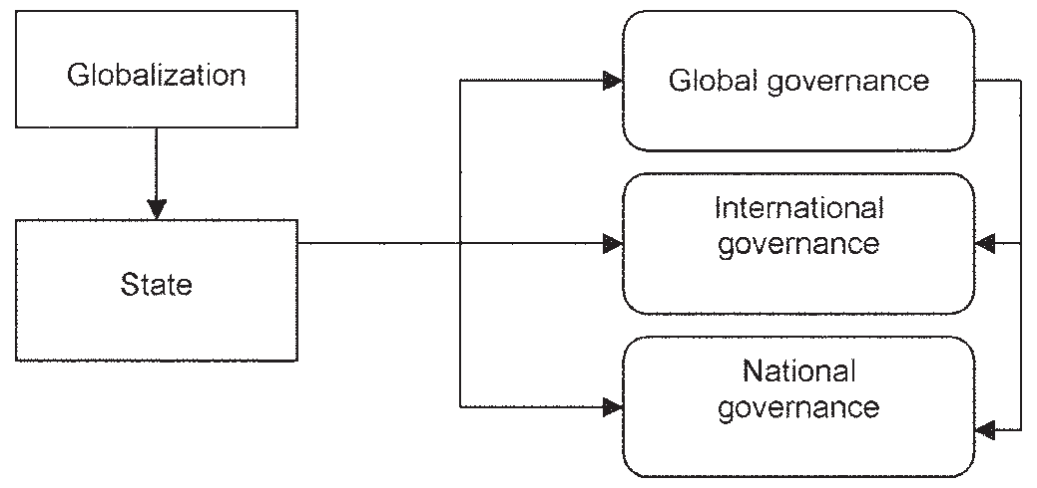
Governance responses to globalization challenges

International law has different functions within vertical and horizontal strategies on infectious diseases and the three governance frameworks (Table 1). An historical overview of infectious disease governance delineates the strategic emphases in the governance frameworks and the functions of international law.

**Table 1:** Governance frameworks, public health strategies, and international law on infectious diseases

Governance framework	Primary strategic emphasis	Function of international law	Infectious disease example
National	Vertical public health strategies	None	National sanitary reform, 19th century
International	Horizontal public health strategies	Provides architecture for horizontal public health strategies	International Health Regulations
Global	Vertical public health strategies	Provides norms informing vertical public health strategies	Global Fund to Fight AIDS, Malaria, and Tuberculosis

## Horizontal International Regimes and Infectious Diseases, 1851–1951

Before the mid-19th century, states contended with infectious diseases through national governance. States adopted policies to manage infectious disease threats without international cooperation (*10*). The increased volume and speed of international trade and travel moved states from national to international governance in the mid-19th century, and the 1851 International Sanitary Conference marked the beginning of international governance on infectious diseases (*11*). International governance focuses primarily on horizontal strategies concerning the exportation and importation of infectious diseases. In the first century of international health governance, three horizontal international legal regimes relating to infectious diseases appeared—the classical, organizational, and trade regimes.

International sanitary conventions adopted from the late 19th century until World War II (*12*) and the World Health Organization (WHO)’s International Sanitary Regulations (1951) (later renamed the International Health Regulations [IHR]) (*13*) represent the classical regime. The IHR’s purpose—“to ensure the maximum protection against the international spread of disease with minimum interference with world traffic” (*13*)—captures the classical regime’s objectives. This set of rules focuses on transmission of diseases across borders by requiring that 1) states notify other countries about outbreaks of specified diseases in their territories and maintain adequate public health capabilities at points of disease exit and entry; and 2) disease-prevention measures restricting international trade and travel be based on scientific evidence and public health principles (*13*).

The second horizontal international legal regime is organizational, that is, international health organizations created to deal with infectious diseases and other public health problems (*10*). WHO serves as the leading representative of this governance framework. Although international law was central to the creation of international health organizations, the treaties establishing them did not impose specific duties regarding infectious disease control (*14*). States created international health organizations to facilitate horizontal cooperation in public health; however, unlike the classical regime, the organizational regime’s legal duties in regard to infectious disease control are few (*14*).

The third horizontal regime created in the 1851–1951 period was the trade regime, represented by the General Agreement on Tariffs and Trade (GATT, 1947), which liberalized trade but recognized that states may restrict trade to protect health (GATT, Article XX[b]). Trade-restricting health measures are legitimate if the measures conform to GATT rules. Thus, the trade regime contributes to horizontal international governance on infectious diseases.

## Globalization, Infectious Diseases, and Governance, 1951–2002

### Transition from the Classical to the Trade Regime

The classical regime’s effectiveness has long been an issue. From Koch’s criticism of late 19th-century international sanitary conventions (*15*) to analysis in the 1960s and 1970s of the IHR’s problems (*16*–*19*), the classical regime’s contribution to international governance on infectious diseases has been questionable. In the past half-century, the classical regime’s importance has diminished, while the trade regime’s influence has grown. Two events reflect this shift. First, in 1995 WHO recognized that the IHR did not achieve their twin goals of maximum protection from the spread of international diseases while incurring minimum interference with world traffic (*20*). WHO launched an effort to revise the regulations to update the classical regime for new globalization challenges (*21*).

Second, the World Trade Organization (WTO) became the central horizontal regime for international law on infectious diseases after its creation in 1995. The Agreement on Trade-Related Aspects of Intellectual Property Rights (TRIPS), the Agreement on the Application of Sanitary and Phytosanitary Measures (SPS Agreement), and the WTO’s powerful dispute settlement mechanism made WTO more important for infectious disease control policy than the discredited IHR. The trade regime’s ascendancy over the classical regime is apparent in the contrast between the public health attention and controversy generated by WTO agreements and the IHR’s obscurity in global public health discourse.

## IHR Revision: Rejuvenation or Death of the Classical Regime?

The shift from the classical to the trade regime raises questions about the IHR revision process. WHO seeks to rejuvenate the regulations to make the classical regime more effective against contemporary disease threats (*20*–*22*). The IHR revision process may, however, signal the classical regime’s death.

The revised IHR would have the same objectives as the original regulations: maximum protection against the international spread of disease while incurring minimum interference with world traffic (*21*). To date, the revision process has moved away from binding legal rules on disease notifications—one of the classical regime’s pillars—to reliance on global information networks, represented by WHO’s Global Outbreak Alert and Response Network (hereafter, the Global Network). WHO argues that this network has helped the organization identify, verify, and investigate hundreds of outbreaks since 1998, including outbreaks of cholera, meningitis, hemorrhagic fevers, viral encephalitis, and anthrax (*23*). WHO’s Executive Director of Communicable Diseases claims the Global Network operates “within the framework” of the IHR (*23*).

The claim that the IHR support the Global Network is not correct under international law. First, the Global Network collects data from government as well as nongovernment sources. The IHR only authorize WHO to use information provided by member states (*13*,*21*). WHO’s proposals to include in the revised IHR an ability to collect data from nongovernment sources (*21*) demonstrate that the IHR cannot provide the legal foundation for the Global Network’s incorporation of nongovernment information. Second, the IHR only address three diseases—cholera, yellow fever, and plague (*13*). This limited coverage was one reason WHO wanted to revise the regulations (*21*). The IHR cannot support WHO’s ability to manage, through the Global Network, meningitis, hemorrhagic fevers, viral encephalitis, anthrax, and other diseases not subject to the IHR.

In addition, the Global Network operates without the revised IHR being in place. From July 1998 to August 2001, WHO used this network to verify “578 outbreaks of potential international importance in 132 countries, and investigated many hundreds more” (*23*). These statistics suggest that WHO’s global surveillance strategy operates without the IHR revision process being completed. Revising the IHR to support global surveillance—the first raison d’être of the classical regime—does not appear urgently required given WHO’s claims of its Global Network’s success.

WHO’s ideas for strengthening global surveillance under the revised IHR center on requiring member states to report all “public health emergencies of international concern” (*21*) to WHO. Member states would use WHO-developed criteria to assess whether an outbreak constitutes such an emergency. The criteria would include whether the event is serious, unexpected, and likely to involve international spread and to trigger trade and travel restrictions (*21*). The problem is that member states will want to determine for themselves whether a disease event constitutes a “public health emergency of international concern,” no matter how many useful criteria WHO provides.

Further, the criteria are subjective rather than objective (e.g., is a disease event serious?), leaving member states with the discretion to argue that they did not report an event because they did not believe the event met the criteria. The fact that WHO reaches a different conclusion would not trigger legal consequences for the member state that failed to report an event. WHO’s proposals on reporting “public health emergencies of international concern” do not indicate whether the criteria will be exclusive and legally binding or whether member states can use different criteria, which they develop.

The move from specific disease reporting to reporting “public health emergencies of international concern” indicates that WHO seeks to improve surveillance on major disease events rather than routine outbreaks. As the Global Network suggests, WHO will likely initially learn about major infectious disease events through sources other than member-state notifications. The Global Network approach reduces the legal importance of official member-state reports concerning major disease events.

WHO has also proposed that the revised IHR allow member states to make confidential, provisional notifications (*21*). WHO claims that the IHR do not allow such notifications (*21*). Concerning those diseases subject to the regulations, WHO’s claim is accurate; however, the IHR cover only three diseases. WHO member states have always been free to consult with WHO staff about diseases not subject to the IHR. This suggested change only affects outbreaks of cholera, plague, and yellow fever, not the majority of infectious diseases considered global threats.

WHO has also proposed improving maximum protection against the spread of international diseases by requiring member states to have national surveillance systems that meet minimum requirements, including the ability to identify “public health emergencies of international concern” (*21*). Given the history of IHR violations and member-state reluctance to limit sovereignty, member states are unlikely to bind themselves to minimum standards. Further, WHO’s proposals contain no discussion of what happens when member states fail to meet those standards.

Even if WHO member states agreed to minimum standards for national surveillance, the requirements would be empty without the commitment of industrialized countries to fund surveillance improvements in unindustrialized countries. Unindustrialized countries would oppose a legal requirement to improve national surveillance without financial commitment from industrialized countries. The end result would likely be authorization for WHO to issue recommendations on how member states should organize national surveillance. WHO already possesses, however, the power to issue recommendations (WHO Constitution, Article 23).

The IHR’s second objective seeks maximum protection against international disease spread with minimum interference with world traffic (*13*). The existing IHR contain maximum measures that member states may take against trade and travel with respect to cholera, plague, and yellow fever (*13*). The IHR did not prevent WHO member states from imposing excessive and irrational travel- and trade-restricting health measures. Under WHO’s proposals, the revised IHR would empower WHO to make recommendations about how member states should handle “public health emergencies of international concern” (*21*).

WHO’s proposals mention “recommendations,” which presumably would not be legally binding. WHO also proposes, however, that a core obligation of the revised IHR be that member states apply measures recommended by WHO during public health emergencies of international concern (*21*). Why WHO confuses obligations with recommendations is not clear. If history is any guide, member states will not allow WHO to issue binding regulations on an ad hoc basis.

Constitutionally, World Health Assembly approval would be required before any rule becomes binding (WHO Constitution, Article 21). This requirement, supported by member states’ guarding of sovereignty, means the revised IHR would only allow WHO to make recommendations about how member states should handle public health emergencies of international concern. Such authority would be redundant because WHO already has the power to issue recommendations (WHO Constitution, Article 23).

Thus, in connection with minimum interference with world traffic, the revised IHR would replace legally binding requirements with the authority to issue nonbinding recommendations, a power WHO already has. This shift does not address what happens when member states ignore WHO recommendations. The existing problem of member states’ behaving in irrational, unjustified ways against outbreaks in other countries is left unresolved.

Today, WTO provides the more important forum for states concerned about irrational trade-restricting health measures because of the SPS Agreement and the WTO dispute settlement mechanism. Thus, the second raison d’être of the classical regime—disciplines against irrational health measures—has weakened for travel-related measures and migrated to the trade regime for health measures that restrict trade in goods.

## Vertical International Governance

In addition to the shift from the classical to the trade regime, new international governance frameworks focusing on vertical public health strategies were developed in the post-1945 period. These approaches seek to reform how a government deals with its health inside the state’s territory.

The “soft-law regime” represents guidelines, practices, and policies generated by international health organizations for adoption by states. Such norms are not legally binding, which is why they constitute soft rather than hard law. WHO has generated many soft-law norms. In fact, WHO has preferred soft law to the creation of binding legal commitments (*24*). The horizontal organizational regime has thus proved more valuable for creating vertical public health strategies than for providing discipline in interstate public health relations.

The “environmental regime” encompasses international environmental law, much of which seeks to reduce environmental threats to human health (*25*,*26*). Environmental treaties often require states to reduce environmental degradation within their territories and through cross-border transmission of harmful products. International environmental law supports both horizontal and vertical strategies. Such law is, however, weakest in connection with vertical public health strategies because it does not address local air and water pollution, the major environmental cause of illnesses and deaths due to infectious diseases (*27*).

The “human rights regime” imposes obligations on governments for treatment of persons in their territories. International human rights law is almost entirely vertical in orientation. Although such law has long incorporated public health, the HIV/AIDS pandemic brought international human rights law to bear more prominently on public health (*28*). Public health experts argue that international human rights law protects persons living with HIV/AIDS from discrimination and imposes obligations on governments to respect, protect, and fulfill their citizens’ human right to health by making prevention and treatment programs universally available (*29*). International human rights law contributes to vertical strategies that seek to control infectious diseases within states rather than address their cross-border movement.

## Global Governance Mechanisms

The third major change of the post-1945 period is development of global governance mechanisms. Global governance involves states, international organizations, and nonstate entities, such as multinational corporations and nongovernment organizations. Nonstate actor involvement distinguishes global from international governance (*8*). Nonstate organizations have long been involved in national and international governance (*30*), but globalization has stimulated new forms of governance in which such organizations have heightened roles. The effectiveness of horizontal international governance through the organizational regime became a major issue in the late 20th century. Although WHO eradicated smallpox, the organization has been ineffective in handling the increasing global devastation wrought by emerging and reemerging infectious diseases, especially HIV/AIDS, malaria, and tuberculosis. The organizational regime’s infectious disease problems have encouraged exploration of new approaches.

Public health experts increasingly focus on global health governance (*8*,*31*–*34*), with emphasis on the role of nonstate organizations. The best examples of this trend are public-private partnerships, which have proliferated in global public health (*35*). Nonstate actor participation in global governance efforts ranges from the formal to the informal. Nongovernment organizations’ presence on the governing body of the Global Fund to Fight AIDS, Malaria, and Tuberculosis (Global Fund) represents formal nonstate participation in global governance (*36*). WHO’s use of information from nongovernment sources in the Global Network informally incorporates nonstate entities. Global governance mechanisms seek to provide “global public goods” for health that states, especially unindustrialized countries, can use within their territories to reduce the prevalence of infectious disease. Such global governance mechanisms primarily support vertical public health strategies.

## The Access Regime

Many global governance initiatives work to increase access to drugs, vaccines, and other medicines. Public-private partnerships—such as Global Alliance for Vaccines and Immunization, Global Alliance on TB Drug Development, and Medicines for Malaria Venture —seek to develop and/or deliver more effectively new or existing drugs and vaccines in unindustrialized countries. The Global Fund hopes, for example, to increase access to antiretrovirals in unindustrialized countries (*36*). The global movement to increase access to essential medicines involves the evolution of a new governance framework—the so-called access regime—that has become the most prominent development in international law on infectious diseases.

The access regime arose from the clash of the horizontal trade regime and the vertical human rights regime. The core of this clash was the collision of the TRIPS-led movement for greater protection for patented pharmaceutical products with the human rights-inspired effort to increase access to essential medicines. The “TRIPS versus public health” battle produced a dramatic moment in November 2001, when WTO adopted the Declaration on the TRIPS Agreement and Public Health (*37*). The Declaration places public health objectives, especially access to medicines, above the trade-related goal of increasing pharmaceutical patent protection. Experts see the Declaration as a victory for the human right to health and for global health governance (*38*).

The involvement of nonstate entities, namely multinational pharmaceutical corporations and nongovernment organizations (e.g., Médecins Sans Frontières) characterizes the development of the access regime. Nonstate organizations play important roles in all aspects of the access regime, particularly through public-private partnerships for developing new drugs (e.g., Medicines for Malaria Venture, Global Alliance on TB Drug Development) or improving access to existing drugs (e.g., Global Alliance for Vaccines and Immunization, Green Light Committee on second-line tuberculosis drugs [*39*]). Nongovernment organizations’ formal governance role in the Global Fund provides another indication that the access regime represents an important development in global governance on infectious diseases. Finally, governance efforts to improve access to drugs and vaccines are found within each governance framework (Table 2).

**Table 2:** The access regime and governance frameworks^a^

National governance	International governance	Global governance
NGO lawsuits filed in national court systems to force national governments to increase access to HIV/AIDS therapies under the human right to health (e.g., South African case of *Treatment Action Campaign v. Minister of Health* (July 2002))	Unindustrialized-country and WHO advocacy to strengthen the public health safeguards in TRIPS to ensure access to affordable drugs and medicines (e.g., Declaration on the TRIPS Agreement and Public Health)	NGO activism directed at MNCs, international organizations, and national governments (e.g., MSF’s global campaign opposing pharmaceutical MNCs’ lawsuit against South Africa)
		Involvement of MNCs and NGOs in drug-development PPPs
		Formal governance roles for nonstate actors in new institutions (e.g., Global Fund)

^a^NGOs, nongovernment organizations; WHO, World Health Organization; TRIPS, the Agreement on Trade-Related Aspects of Intellectual Property Rights; MNCs, multinational corporations; MSF, Médecins Sans Frontières; PPPs, public-private partnerships;

The access regime takes the human right to health, developed originally as vertical international governance, into the realm of global governance through the leadership of nonstate organizations. The access regime aims to develop not only governance conducive to improved access but also new pharmaceutical products that governments, international institutions, and nonstate organizations can use to reduce illness and death caused by infectious diseases.

The access regime captures how international law’s function differs in global governance from its role in international governance. Norms found in international law, principally the human right to health, inspire global governance on access; however, international law does not provide the architecture for such governance. Public-private partnerships are not based on treaties. The participation of states and international organizations in these global governance efforts is nonbinding under international law. The access regime uses international law differently than states and international organizations have used it for public health since international health diplomacy began in 1851.

Although many view the access regime as progressive, concerns exist about its impact on public health. The emphasis on access may divert attention and resources away from strengthening overall public health and infectious disease control infrastructures. The human rights focus may heighten concern for individual healthcare at the expense of protecting population health. Finally, increasing drug access may exacerbate antimicrobial resistance if proper attention to rational use is lacking (*40*).

## Conclusion

Globalization creates infectious disease challenges that force states to cooperate. Historically, international law has been important in facilitating such cooperation. International law’s use in infectious disease control has to address the governance challenges that globalization presents public health. The last 50 years, and the 1990s in particular, have witnessed shifts in how international law factors into global infectious disease policy. Within the traditional realm of horizontal international governance, attention has moved from the classical to the trade regime, changing the structure and substance of international law’s role in infectious disease control. In addition, revision of the IHR signifies the death of the classical regime rather than its rejuvenation. Such a death would mark the end of the traditional use of international law for infectious disease control. Attention has also shifted from horizontal international governance to vertical global governance. This shift finds international law’s traditional function in international governance supplemented by a context in which international law informs global governance endeavors that are not legally binding.

These developments mean that international law’s role in infectious disease control today has never been more important and uncertain. International law remains important to horizontal international governance, as indicated by WTO’s international legal role in public health. International law also informs vertical global governance even though these regimes find no formal expression in international law. Uncertainty looms, however, in the IHR revision process, questions about WTO’s impact on public health, and concerns that vertical global governance will not deliver global public goods for health in an important and sustainable manner.
